# Urinary Incontinence and Alzheimer’s Disease: Insights From Patients and Preclinical Models

**DOI:** 10.3389/fnagi.2021.777819

**Published:** 2021-12-17

**Authors:** Sarah N. Bartolone, Prasun Sharma, Michael B. Chancellor, Laura E. Lamb

**Affiliations:** ^1^Department of Urology, Beaumont Health, Royal Oak, MI, United States; ^2^Oakland University William Beaumont School of Medicine, Rochester Hills, MI, United States

**Keywords:** Alzheimer’s disease, urinary incontinence, aging bladder, neurourology, muscarinic receptors

## Abstract

Alzheimer’s disease effects a large percentage of elderly dementia patients and is diagnosed on the basis of amyloid plaques and neurofibrillary tangles (NFTs) present in the brain. Urinary incontinence (UI) is often found in the elderly populations and multiple studies have shown that it is more common in Alzheimer’s disease patients than those with normal cognitive function. However, the link between increased UI and Alzheimer’s disease is still unclear. Amyloid plaques and NFTs present in micturition centers of the brain could cause a loss of signal to the bladder, resulting in the inability to properly void. Additionally, as Alzheimer’s disease progresses, patients become less likely to recognize the need or understand the appropriate time and place to void. There are several treatments for UI targeting the muscarinic and β3 adrenergic receptors, which are present in the bladder and the brain. While these treatments may aid in UI, they often have effects on the brain with cognitive impairment side-effects. Acetylcholine esterase inhibitors are often used in treatment of Alzheimer’s disease and directly oppose effects of anti-muscarinics used for UI, making UI management in Alzheimer’s disease patients difficult. There are currently over 200 pre-clinical models of Alzheimer’s disease, however, little research has been done on voiding disfunction in these models. There is preliminary data suggesting these models have similar voiding behavior to Alzheimer’s disease patients but much more research is needed to understand the link between UI and Alzheimer’s disease and discover better treatment options for managing both simultaneously.

## Introduction

It is estimated that there are 35 million people living with dementia globally and by the year 2030, that number is predicted to double (Lee et al., 2017). Alzheimer’s disease (AD) accounts for more than 80% of elderly dementia patients (Jung et al., 2017). AD is a chronic, progressive neurological disease that evolves through stages of cognitive and psychomotor skill decline (Gaugler et al., 2014). AD diagnosis is based on the presence of protein aggregates found in the brain, including amyloid plaques and neurofibrillary tangles (NFTs), which are also referred to as neurologic lesions (Hall and Roberson, 2012). The progression of AD tends to be slow and progresses over many years through five stages: preclinical, mild cognitive impairment, and mild, moderate, and severe AD dementia (Davis et al., 2018). However, symptoms of AD can occur years before diagnosis and with disease progression (Atri, 2019).

Urinary incontinence (UI) is defined by the International Continence Society as “the complaint of any involuntary leakage of urine” (Abrams et al., 2003). Age-related changes in the genitourinary system often lead to UI, making it a common issue amongst the elderly (Davis et al., 2020) and often coexists in patients with AD (Lee et al., 2014). The association of UI in individuals with dementia increases with progression and severity of cognitive impairment (Orme et al., 2015). Primarily, UI is described in AD patients. While other lower urinary tract symptoms (urgency, frequency, nocturia, pain, dysuria, discomfort) may exist, many AD patients with severe dementia may have limited ability to communicate and possibly even understand medical symptoms. Furthermore, these symptoms are subjective and do not necessarily have easy, non-invasive ways to measure. Therefore, even though these symptoms have not been described in the literature to be associated with AD, we cannot rule out that they do not exist within this population due to our poor ability to assess these subjective and often descriptive symptoms. For the purpose of this review, we will focus on UI and no other lower urinary tract symptoms as UI is the most common presentation in AD (Lee et al., 2014) and what has been most studied within this population.

Bladder dysfunction is the costliest lower urinary tract disorder and one of the top three negative effectors on quality-of-life, behind stroke and AD (Coyne et al., 2009). Independently, both UI and dementia increases the likelihood of placement in a nursing home (Orme et al., 2015). Of patients in nursing homes on Medicare, 64% of them have AD or related dementia (Gaugler et al., 2014). UI has been found to be more common in patients with dementia than those normal cognitive function (Lee et al., 2017); 83% of nursing home residents with UI have dementia compared to 58% of residents with normal bladder function (Ouslander et al., 1990). Additionally, it is expected that UI is underreported in the AD population due to forgetfulness, embarrassment, or denial of the condition (Skelly and Flint, 1995). A study by Sakakibara et al. (2008) found that nursing home residents with dementia were more likely to develop UI during 12-month follow-up period and UI was less likely to resolve compared to residence without dementia. Interestingly, the likelihood of someone with dementia developing UI is greater when institutionalized compared to receiving home care (Orme et al., 2015). A 1994 study in Sweden showed that UI prevalence in dementia patients is 74% in nursing homes and 32% at home (Hellström et al., 1994), suggesting the difference in environment and care of AD patients may play a part in development of UI. However, the underlying association connecting UI and AD is unknown, as is whether AD leads to bladder dysfunction, or if bladder dysfunction precedes the onset of AD symptoms.

## Behavioral Effects of Alzheimer’s Disease on Urinary Incontinence

Multiple studies have reported that AD patients are aware of the need to urinate, however, are unable to make it to a bathroom prior to voiding, which is known as functional UI (Sakakibara et al., 2008; Lee et al., 2014). Functional UI is incontinence associated to cognitive decline, with no pathology in the lower urinary tract, and is often seen in patients with AD as a result of cognitive disability and decreased motivation and mobility (Sakakibara et al., 2008; Lim, 2017). Causes of UI are complex and not yet completely understood as many aspects outside of the lower urinary tract, such as psychological factors like anxiety and depression, play a role in its progression (Bogner et al., 2011). As AD progresses, patients decline in both cognitive and physical abilities such as memory loss, changes in personality, confusion, and inability to complete daily tasks such as toileting (Jung et al., 2017). These progressing impairments caused by AD can lead to an increasing risk of UI as it is difficult to identify when or where to void and patients’ ability to access the toilet becomes difficult (Lee et al., 2017). It is challenging to accurately assess UI in patients with cognitive and physical deterioration. Often, the diagnosis of UI involves invasive urodynamic studies, however, these are problematic to obtain in the AD population as patient cooperation and compliance is difficult (Lim, 2017). By using bladder health history and validated urinary symptom questionnaires, we can learn about UI in AD patients, however, the application of these questionnaires is limited as it is often difficult for AD patients to recall timelines and events of UI (Lee et al., 2014). While a portion of UI can be attributed to function UI due to cognitive decline, there are other physiological factors that link AD to UI.

Fecal incontinence is prevalent in the elderly but like UI, little is known about the physiological connection between them. One study found dementia as an independent risk factor for significantly increased likelihood fecal incontinence (Nakanishi et al., 1997). The only treatments used for fecal incontinence is increased fiber intake, laxatives, enemas, and increasing quality of care to ensure a toilet schedule is followed (Leung and Rao, 2009).

## Damage to Micturition Centers in the Brain

The ability to void is controlled by a multitude of signals carried between the bladder and the brain, specifically the frontal cortex, basal ganglia, and pontine micturition center (PMC), or Barrington’s nucleus, located in the dorsal pons. As the bladder fills, the PMC receives signal through central nervous system (CNS), the signal is then sent through the peripheral nervous system to the bladder to promote micturition. During the storage phase, the basal ganglia and frontal cortex inhibit contraction by inhibiting parasympathetic activation and active relaxation of the detrusor muscle (Ginsberg, 2013). In addition to the autonomic signals between the brain and bladder, the CNS plays a role in bladder contraction and voluntary release of urine when acceptable (i.e., when on a toilet). This conscience decision to void requires coordination with the relaxation of the internal sphincter, controlled by efferent input from autonomic nervous system, and the external sphincter, controlled by the somatic nervous system through the PMC (Lim, 2017; Tish and Geerling, 2020).

Neurogenic bladder incontinence is most often connected to another neurologic condition. Neurologic lesion in nerve centers of the micturition pathway can lead to neurogenic bladder incontinence. The most common conditions that experience UI are Alzheimer’s disease, birth defects of the spinal cord, brain or spinal cord tumors, cerebral palsy, multiple sclerosis, Parkinson’s disease, traumatic brain injury, and spinal cord injury (Ginsberg, 2013; Tish and Geerling, 2020). Less common cause of neurogenic bladder incontinence are autonomic neuropathy due to diabetes mellitus, pelvic surgery complication, and cauda equina syndrome (Ginsberg, 2013). This type of bladder dysfunction is dependent on the location of the lesions. For example, if the lesion is caused by stroke or brain tumor above the PMC, there is usually reduced awareness of bladder fullness. A spinal cord injury or multiple sclerosis can cause lesions between the PMC and sacral spinal cord (Dorsher and McIntosh, 2012). These lesions alter the signal sent between the brain and bladder, resulting in dysfunction. Similarly in AD patients, damage to the brain occurs in more than one location so pinpointing the region(s) causing UI is elusive (Tish and Geerling, 2020).

Neurologic lesions, either plaques, NFTs, or both, are found in the anteromedial frontal cortex, the center of micturition reflex, in patients with AD (Lee et al., 2017). A functional image study in AD patients demonstrated that the prefrontal cortex is activated when patients had a strong sensation to void (Lee et al., 2017). This study suggests that while the urge to urinate still exists in AD patients, lesions in the frontal cortex may be causing a loss of inhibitory signal to the bladder, which may drive UI (Lee et al., 2017). Neurologic lesions above the brain stem, as is seen in AD, causes a sense of urgency to void the bladder (Yokoyama et al., 2002). A study quantifying NFTs in the pons of AD patients found that NFTs are present and increase with progression of AD (Uematsu et al., 2018). These lesions affect the inhibitory influence of the micturition reflex, resulting in involuntary contraction of the bladder’s detrusor muscle, which can lead to UI (Lee et al., 2014). This would suggest that in patients with AD, lesions in the micturition reflex are leading to involuntary contractions of the bladder, suggesting a possible mechanism for UI. Due to the wide variation in pathophysiology of neurogenic bladder, treatment and medications are guided based on which section of the micturition pathway is affected (Dorsher and McIntosh, 2012).

## Treatments

There are currently several treatment options available for UI in AD patients including: antimuscarinics, β_3_ adrenergic receptor agonists, Onabotulinumtoxin type A (Botox), and non-drug treatment therapies (Hersh and Salzman, 2013). Anti-muscarinics are often the first line treatments for bladder symptoms including urgency, frequency, and incontinence. These include oxybutynin, tolterodine, darifenacin, slifenacin, trospium, and fesoterodine (Table 1; Abrams and Andersson, 2007). Antimuscarinics work by antagonizing muscarinic acetylcholine receptors (mAChRs). M_3_ subtype of mAChR act as the primary mediators of contractions in bladder detrusor muscles by binding to acetylcholine molecules. Many studies have shown patients taking an antimuscarinic have decrease adverse urinary episodes (frequency, incontinence) as measured by an increase in quality-of-life through a short form patient satisfaction survey (Burgio et al., 2006; Lim, 2017). However, not all antimuscarinics used to treat UI are selective for M_3_ receptors alone. For example, oxybutynin non-selectively targets both M_1_ and M_3_ receptors. M_1_ receptors are widely expressed in the CNS (Jiang et al., 2014). Non-selective anti-muscarinic can easily cross the blood-brain barrier, allowing them to act on the M_1_ receptors in the cerebral cortex and hippocampus (Bogner et al., 2011). CNS side effects of antimuscarinics may range from mild symptoms of headache and dizziness to severe cognition impairment and even coma (Gulsun et al., 2006). Due to the non-selective nature of many antimuscarinics, they are contraindicated in conditions like myasthenia gravis, acute asthma, myocardial infarction and hyperthyroidism, to name a few (Naji and Gatling, 2021). The use of drug therapies like darifenacin that selectively target the M_3_ receptor would be ideal choice of an antimuscarinic, as the chance of adverse CNS reaction could be minimized (Chancellor and Boone, 2012).

**TABLE 1:** Potential drug treatments of Alzheimer’s disease and urinary incontinence.

Drug class	Drug name	MOA	Action site	Indications
**Anti-muscarinic**	Oxybutynin Tolterodine Solifenacin	Blocks muscarinic receptors from binding to Ach. Ach is released from parasympathetic nervous system and stimulates detrusor muscle for contraction	M1-M3 M2-M3 M3	Decreased bladder spasms and urgency in overactive bladder incontinence
	Darifenacin		M3	Urinary urgency, urge incontinence, urinary frequency, and/or nocturia due to overactive bladder
**Acetylcholinesterase inhibitor**	Donepezil Rivastigmine Galantamine	Reversibly binds to AChE and inhibits the hydrolysis of ACh	AChE	Alzheimer disease
**β_3_ agonist**	Mirabegron (Myrbetriq)	Increases the bladder storage capacity through relaxation of detrusor smooth muscle during the storage phase of the fill-void cycle of the urinary bladder	β_3_	Urinary urgency, urge incontinence, urinary frequency, overactive bladder
**Desmopressin**	Ddavp	Synthetic form of Anti diuretic hormone that increases water reabsorption at the distal convoluted tubule reducing frequent urination	Vasopressin V_2_	Hemophilia A, Von Willebrand disease, Sleep enuresis, Nocturia

In contrast, mild to moderate AD is commonly treated by Acetylcholinesterase (AChE) inhibitors like donepezil, rivastigmine and galantamine (Table 1). Donepezil hydrochloride, rivastigmine tartrate and galantamine hydrobromide are central AChE inhibitors that decrease degradation of acetylcholine, thus increasing the concentration of acetylcholine in the synaptic cleft. These agents inhibit AChE selectively in the brain, reversing cognitive decline in Alzheimer’s disease patients (Sakakibara et al., 2008). Despite selectively inhibiting AChE in the brain, AD patients taking AChE inhibitors were found to have more instances of UI diagnosis than non-AD patients (Gill et al., 2005). This suggest that this could be due to longer availability of acetylcholine at PMC, which has the central control of detrusor muscle (Lim, 2017).

Anti-muscarinic and AChE inhibitors have directly opposing drug-drug interactions, posing a particular challenge in managing UI in AD patients. While AChE inhibitors can cause UI symptoms, antimuscarinics can reduce the efficacy of AD pharmaceutical therapies (Gill et al., 2005). Although there are international guidelines for management of UI put forth by the International Continence Society (Abrams et al., 2017), there is little consideration for the implications of this management for patients with co-morbidities (Orme et al., 2015). Patients with AD have higher opportunity to have comorbidities and are more likely to be prescribed medications that induce urge incontinence (Lee et al., 2017). Previous studies have found that AD patients receiving AChE inhibitors were also more likely to be prescribed an anti-muscarinic (Roe et al., 2002). This can further complicate the diagnosis because UI present in AD patients may be a side effect of AChE inhibitors (Gill et al., 2005).

In 2015 American Geriatrics Society Beers Criteria included antimuscarinics as one of the inappropriate medications that older adults should avoid (American Geriatrics Society 2015 Beers Criteria Update Expert Panel, 2015). A longitudinal study from National Institute on Aging found 23% of participants over the age of 65 developed dementia in the 7 years period, that they were prescribed antimuscarinics for overactive bladder (OAB). Of the 23%, 80% developed AD (Risacher et al., 2016). Another study published in *The British Medical Journal* found that participants that received anticholinergic were 11% more likely to develop dementia (Richardson et al., 2018). The risk of dementia was 30% more likely in the same group of participants that were prescribed medications with higher anticholinergic effect (Richardson et al., 2018). Additionally, the compliance rate of patients on antimuscarinic for OAB is as low as 8%, due to the combination of side effects, unmet treatment expectations as well as cost (Chancellor et al., 2013).

UI management should be individualized in AD patients and reviewed frequently to ensure the use is effectively treating UI without adverse cognitive effects (Orme et al., 2015). Patients prescribed donepezil along with anticholinergic were found to have significantly worsening in serial cognitive assessment relative to patients solely being treated with donepezil. Similarly, the more appropriate treatment to minimize UI symptoms among AD patients may be to reduce the dose of AChE inhibitors rather than adding anti-muscarinic (Gill et al., 2005). An alternative to AChE inhibitors is memantine, an N-methyl-D-aspartate (NMDA) glutamate receptor antagonist which was found to reduce clinical deterioration in moderate to severe AD. However, it is difficult to compare the effects of memantine to AChE inhibitors because studies regarding AChE inhibitors include less severe AD patients. In contrast, memantine studies have included late stage AD patients (Reisberg et al., 2003) and UI is not listed as one of the side effects of memantine but urinary tract infection and acute renal failure are (Kuns et al., 2021). β_3_ adrenergic receptor agonists like Mirabegron for OAB are emerging as a better option to antimuscarinics. In certain cases symptoms management of nocturia due to nocturnal polyuria in adults who awaken at least 2 time per night to void through medications like desmopressin could be more effective (Chancellor and Watanabe, 2019). It would be important to remember that desmopressin can cause hyponatremia and serum sodium must be normal before starting and rechecked at 1 and 4 weeks after starting desmopressin for this indication of UI.

Multiple non-drug treatment/therapy options are also available for UI management in AD patients. Scheduled toileting or prompted voiding regiments is one therapy in which patients are toileted on a schedule (usually every 2 h). These patients have been shown to reduce UI by an average of 32% and appear to be a beneficial approach in managing UI in some patients (Skelly and Flint, 1995). One study found behavioral treatment (80.7%) was more effective at managing urge incontinence than placebo (39.4%) in randomized controlled trials (Burgio et al., 2006). Pelvic floor therapies and electrical stimulation are often used as a non-drug therapy option in patients with UI. However, these therapies were found to be unsuccessful in AD patients with UI as the AD patients were unable to cooperate with the therapy (Tobin and Brocklehurst, 1986) and became agitated with the procedure (Lamhut et al., 1992). Lamhut et al. (1992) found that there was a 20% increase in incontinence episodes in AD patients that underwent electrical stimulation therapy. These findings could suggest that the UI experienced by these patients is primarily due to functional impairments and not a bladder centric dysfunction (Lamhut et al., 1992).

## Studying Urinary Incontinence in Alzheimer’s Disease Pre-Clinical Models

To date, there are 206 rodent models available for AD research which include transgenic, natural, and intervention models (Li et al., 2019; Lamb et al., 2020). Non-primate mammals do not develop the same hallmarks of AD as seen in humans (i.e., NFTs, amyloid beta plaques), however, they do develop other forms of tauopathies and senile plaques (Gołaszewska et al., 2019). These animals display Alzheimer’s-like diseases (ALD), mimicking many of the clinical symptoms of AD (Gołaszewska et al., 2019). To study human AD pathology in animal models, several transgenic rodents were developed by inserting human tau and amyloid beta, resulting in the development of human NFTs and plaques (Lamb et al., 2020).

While there has been extensive research done on AD in these models, few studies have investigated bladder function. One AD model to report altered bladder function is the APP^SL^/PS1^M146L^ model (Swedish/London-FAD mutations) (Köhler et al., 2005). This model is one of the most commonly used AD preclinical models. Biallosterski et al. (2015) investigated voiding patterns of the APP/PS1 mice compared to age-matched, C57BL/6NCrl control mice. They found the APP/PS1 mice urinate smaller volumes in more central areas of the cage (Biallosterski et al., 2015). The authors postulate that the smaller volumes could be attributed to behaviors changes as well as increased afferent activity in the lower urinary tract, although this mechanism is still unclear (Biallosterski et al., 2015). Further, it was thought that the voiding in the center area of the cage rather than corners was due to increased locomotor activity due to anxiety. The APP/PS1 mice display other anxiety-indicative behaviors including spending significantly less time in the open arms of an elevated zero maze task (Biallosterski et al., 2015). In rodent models, urinating in the center of the cage is abnormal and often indicates a psychological or physiological defect (Hill et al., 2018), both of which are known to be present in AD. Similar anxiety prone behaviors have also been reported in individuals with AD (Bogner et al., 2011). One study found persons diagnosed with an anxiety disorder suggests an increased risk of UI later in life (Bogner et al., 2011). These findings attributed UI to a functional loss associated with AD (Bogner et al., 2011).

As previously discussed, muscarinic receptors are present in both brain and bladder tissue and are targets for UI and AD treatments. Muscarinic receptors act as autoreceptors, which regulate ACh release in CNS. Presynaptic M_2_ receptors act as negative autoreceptors and can decrease ACh release. One study in M_2_ and M_4_ receptor KO mice saw increased levels of hippocampal released ACh, which may be associated with cognitive deficits (Tzavara et al., 2003). While this model may not be an AD preclinical model, it could be used to better understand the role muscarinic receptors play in AD and the effect of muscarinic targeting drugs on the brain and bladder. Additionally, one study looked at mutated APP overexpressing familial AD mouse model (PDAPP) and found they have reduced hippocampal ACh release and regulation compared to control mice (Bales et al., 2006). Aβ plaques present in the PDAPP interact with choline transporter, which impairs ACh release and synaptic transmission (Bales et al., 2006). PDAPP mice had a similar profile of ACh release dysregulation as the M2 KO model (Tzavara et al., 2003; Bales et al., 2006) as the influence on muscarinic inhibitory receptors leads to inefficient regulation of synaptically released ACh. Additional research on these models could give a better insight to effects of ACh dysregulation on UI and AD.

A magnitude of research has been done in pre-clinical models to discover the effects of AD and UI treatments on the muscarinic and AchE receptors. In ddY mice on an oral treatment of Darifenacin, there was a 22-fold higher potency at M3 than M2 receptors, making it a good target for the bladder (Yamada et al., 2006). Darifenacin acts quickly and is long-lasting (at least 12-h in ddY mice) (Yamada et al., 2006). These experiments should be done in the available AD mouse models to determine if there is an effect on cognition and neurologic lesions. When treating Tg2576 mice, Donepezil was found to reduce AB plaque number and increase synaptic density (Dong et al., 2009). Administration of Rivastigmine directs the processing of the APP protein toward the non-amyloidogenic pathway, with less alpha-secretase activity, which cleaves APP to toxic AB plaques (Ray et al., 2020). No studies have been done in mouse models to explore the effects of these AD drugs on the muscarinic receptors in the urinary bladder and how they alter bladder function.

Given the lack of investigation into bladder function in AD mouse models, an alternative approach is to examine mouse models with dementia and/or advanced aging phenotypes. Again, there is a large gap in understanding bladder function in these models as well. Two accelerated aging models that did look at bladder function were the SAMP8 and Klotho deficient models. SAMP8 is a senescence-accelerated model that exhibits an accelerated aging phenotype. SAMP8 mice have increased number of voids, increased neurogenic contractility, a slight acetylcholine sensitization, and greater urethral excitation with no change in cholinergic nerve density (Triguero et al., 2014). Bladder dysfunction has also been tied to the α*Klotho* (KL) hypomorph accelerated aging model (Bartolone et al., 2020). Mice lacking expression of klotho protein had an increase number of voids with decreased volume per void, measured by void spot assay (Bartolone et al., 2020). In mice with heterozygous expression of the α*Klotho* gene, there was a decrease in the levels of tau in the cerebral spinal fluid, which was protective against cognitive changes in older mice (Driscoll et al., 2021). With the limited urinary bladder data in many of the available AD rodent models, it is difficult to assess whether these models effectively recapitulate what is seen in human patients. While the above findings are in line with what it seen in some AD patients, more studies are needed.

## Discussion

UI comorbidity among AD patients is a common occurrence. The likelihood of UI existing or developing in AD patients is greater than that of the elderly population without cognitive decline (Lee et al., 2017). However, the underlying causes of this association are still not well understood. Studies suggest UI could be caused by behavioral and functional loss due to neurologic lesions in the brain. Lesions in the brain, specifically the frontal cortex, basal ganglia, and PMC, could directly modify the micturition reflex leading to UI. Indirectly, UI can be due to neural lesions elsewhere that lead to memory loss, anxiety, and confusion. These effects make it difficult for AD patients to recognize the need to void and the appropriate time and place to void, leading to a diagnosis of UI. Additionally, UI could be due to the negative effects of AChE inhibitors, which are common pharmacotherapy for AD management. While these treatments are effective in the treatment of AD symptoms, they also have direct negative effects on the PMC and bladder receptors increasing the likelihood of UI (Gulsun et al., 2006).

It is well known that there is a dysregulation in brain-bladder communication that occur in AD patients. To better understand the mechanism between AD and UI it is crucial to look at pre-clinical models. There are 206 AD rodent models available, yet little research has been done in these models to investigate UI. Data from UI studies in APP^SL^/PS1^M146L^, SAMP8, and KL models report a change in voiding behavior that could be linked to cognitive decline, however, more studies are needed. Additional models, such as muscarinic receptor knockout or ACh dysregulation models can also be used to better understand the connection between AD and UI. Several studies must be done to explore the UI function of mice with ALD with and without common anti-muscarinic and AchE inhibitor treatment.

## Author Contributions

SB, MC, and LL conceived of the manuscript. SB and PS provided analysis and interpretation of studies and wrote the original draft of the manuscript. MC and LL provided analysis and interpretation of studies, critical review, commentary, and revision of the manuscript. LL provided project supervision. All authors contributed to the article and approved the submitted version.

## Conflict of Interest

The authors declare that the research was conducted in the absence of any commercial or financial relationships that could be construed as a potential conflict of interest.

## Publisher’s Note

All claims expressed in this article are solely those of the authors and do not necessarily represent those of their affiliated organizations, or those of the publisher, the editors and the reviewers. Any product that may be evaluated in this article, or claim that may be made by its manufacturer, is not guaranteed or endorsed by the publisher.
